# *AtWuschel* Promotes Formation of the Embryogenic Callus in *Gossypium hirsutum*

**DOI:** 10.1371/journal.pone.0087502

**Published:** 2014-01-31

**Authors:** Wu Zheng, Xueyan Zhang, Zuoren Yang, Jiahe Wu, Fenglian Li, Lanling Duan, Chuanliang Liu, Lili Lu, Chaojun Zhang, Fuguang Li

**Affiliations:** 1 State Key Laboratory of Cotton Biology, Institute of Cotton Research, Chinese Academy of Agricultural Sciences, Anyang, Henan, China; 2 Institute of Microbiology, Chinese Academy of Sciences, Beijing, China; USDA-ARS-SRRC, United States of America

## Abstract

Upland cotton (*Gossypium hirsutum*) is one of the most recalcitrant species for *in vitro* plant regeneration through somatic embryogenesis. Callus from only a few cultivars can produce embryogenic callus (EC), but the mechanism is not well elucidated. Here we screened a cultivar, CRI24, with high efficiency of EC produce. The expression of genes relevant to EC production was analyzed between the materials easy to or difficult to produce EC. Quantitative PCR showed that CRI24, which had a 100% EC differentiation rate, had the highest expression of the genes *GhLEC1*, *GhLEC2*, and *GhFUS3*. Three other cultivars, CRI12, CRI41, and Lu28 that formed few ECs expressed these genes only at low levels. Each of the genes involved in auxin transport (*GhPIN7*) and signaling (*GhSHY2*) was most highly expressed in CRI24, with low levels in the other three cultivars. WUSCHEL (WUS) is a homeodomain transcription factor that promotes the vegetative-to-embryogenic transition. We thus obtained the calli that ectopically expressed *Arabidopsis thaliana Wus* (*AtWus*) in *G. hirsutum* cultivar CRI12, with a consequent increase of 47.75% in EC differentiation rate compared with 0.61% for the control. Ectopic expression of *AtWus* in CRI12 resulted in upregulation of *GhPIN7*, *GhSHY2*, *GhLEC1*, *GhLEC2*, and *GhFUS3*. *AtWus* may therefore increase the differentiation potential of cotton callus by triggering the auxin transport and signaling pathways.

## Introduction

Somatic embryogenesis (SE) is a principal model for studying the growth and development of zygotic embryos in higher plants. This process includes callus induction, embryogenic callus (EC) formation, embryo development, and plant regeneration. During the past three decades, much effort has attempted to determine important genes controlling SE [1], [2].

The gene *WUSCHEL* (*Wus*) is essential for stem cell formation and maintenance in shoot and root apical meristems [3], [4]. *Wus* mediates stem cell homeostasis by regulating cell division and differentiation [5]–[7]. In *wus* mutants, apical meristems are unable to preserve the pool of undifferentiated cells [3]. The maintenance function of WUS can be repressed by inducing *AGAMOUS* (AG) expression and floral meristem differentiation [8]. *Wus* was first reported as the key gene promoting SE in *pga6* mutants in *Arabidopsis*, with overexpression of *PGA6*/*Wus* causing the vegetative-to-embryonic transition [9]. *Wus* is crucial for EC renewal during SE in *Arabidopsis* [10]. Overexpression of *Wus* in *Coffea canephora* can also promote SE [11].

Auxin is necessary for SE [12], [13], but the auxin transport and signaling pathways during SE are not well understood. *PIN-FORMED* (*PIN*) genes encoding efflux carrier proteins are involved in auxin transport [14], [15]. Genetic analysis indicates that *PIN1* is a major regulatory factor for auxin gradients in EC and embryo [16]. Auxin regulates auxin-responsive genes via the Aux/IAA (SHY)-ARF module. At sufficiently low auxin concentration, auxin response factors (*ARF*s) are repressed by Aux/IAA. At sufficiently high auxin concentration, Aux/IAA is degraded by SCF^TIR1^, and *ARF*s are activated [17]–[19]. *ARF*s positively or negatively control downstream genes, resulting in responses to auxin signaling. Transcript profiling reveals that the auxin signaling pathway may play a vital role during SE in cotton [13].

*LEC1*, *LEC2*, and *FUS3* are key genes that control SE progression [20], [21]. The capacity for SE is completely repressed in double (*lec1 lec2*, *lec1 fus3*, *lec2 fus3*) or triple (*lec1 lec2 fus3*) mutants of *Arabidopsis thaliana* [21]. *LEC2* expression changes rapidly during auxin responses [22], suggesting that *LEC*/*FUS* may be downstream genes in the auxin signaling pathway [21], [23].

The majority of cotton cultivars are incapable of undergoing SE [24] because of their difficulty in inducing callus differentiation to form EC. Thus, most cultivars are not used for molecular breeding using transgenic technologies with *Agrobacterium*-mediated transformation via SE. Therefore, it is essential to study the mechanism of SE in cotton so as to improve regeneration of various cotton cultivars.

Here, we report the ectopic expression of *A. thaliana Wus* (*AtWus*) in *G. hirsutum* cv. CRI12, a cultivar that shows poor SE ability under established tissue culture methods. *AtWus* promoted differentiation of transgenic callus. Furthermore, ectopic expression of *AtWus* could upregulate *GhLEC1* (*G. hirsutum LEC1*), *GhLEC2*, and *GhFUS3* expression during SE and alter auxin transport and signaling mechanisms. *AtWus* therefore promotes the efficiency of EC differentiation in cotton callus.

## Materials and Methods

### Plant Materials and Tissue Culture Conditions

We selected four cotton cultivars, CRI24, CRI12, CRI41 and Lu28, as experiment materials. CRI24 has a 100% EC differentiation rate and is the main transgenic material used for *Agrobacterium*-mediated method in our lab. CRI12 used to be an important basic breeding material because of traits of its relatively high yield and disease resistance, yet it has a low rate of differentiation during SE. CRI41 and Lu28, the main cultivars planted in China, can not undergo SE because of failure in EC induction.

Seeds of the four cotton cultivars were sterilized with 0.1% (w/v) mercuric chloride for 3 min. The seeds were then washed five times with sterilized distilled water and then germinated on modified Murashige and Skoog (MS) medium (25 g l^–1^ sucrose, 50 ml l^–1^ MSI, 5.6 g l^–1^ agar) for hypocotyl induction. Sterilized seeds were cultured at 28°C with a 14 h/10 h light/dark photoperiod. The hypocotyls from 7-day-old sterile seedlings were cut into 2 cm segments. For transgenic experiments using an *Agrobacterium*-mediated method [25], the hypocotyl cuts of CRI12 were transferred to 250 ml flasks and placed on callus-induction medium (CIM; MS medium plus B5 vitamins, supplemented with 0.05 mg l^–1^ 3-indole acetic acid (IAA), 0.05 mg l^–1^ kinetin, 0.05 mg l^–1^ 2,4-dichlorophexoxyacetic acid, 25 g l^–1^ glucose, 2 g l^–1^ gelrite gellan gum, 50 mg l^–1^ kanamycin, 100 mg l^–1^ cefotaxime, pH 5.8). The medium was changed once per month. After 2 months of culture, all calli were transferred onto EC induction medium (EIM; MSB supplemented with 25 g l^–1^ glucose, 2 g l^–1^ gelrite, 0.5 g l^–1^ MgCl_2_, 0.16 mg l^–1^ kinetin, 0.08 mg l^–1^ IAA, 50 mg l^–1^ kanamycin, 100 mg l^–1^ cefotaxime, pH 6.5). The medium was changed monthly. After 4 months of culture, ECs were transferred to somatic embryo induction medium (SIM; MSB supplemented with 25 g l^–1^ glucose, 2 g l^–1^ gelrite, 0.5 g l^–1^ MgCl_2_, 0.08 g l^–1^ kinetin, 0.12 mg l^–1^ 6-benzylaminopurine, 50 mg l^–1^ kanamycin, 100 mg l^–1^ cefotaxime, pH 6.8), and the medium was refreshed monthly. For non-transgenic experiments, the hypocotyl cut explants of CRI24, CRI12, CRI41, and Lu28 were transferred onto NCIM (CIM lacking kanamycin and cefotaxime), and the medium was changed once per month. After 30 days of culture, all calli were transferred onto NEIM (EIM lacking kanamycin and cefotaxime), and the medium was changed monthly. After 90 days of culture, all calli of CRI24 differentiated into EC, most calli of CRI12 and all calli of CRI41 and Lu28 did not differentiate into ECs. To confirm the lack of capacity for EC differentiation among the latter three cultivars, the calli which can not differentiate in ECs were cultured in NEIM for another 120 days, with the medium being refreshed monthly. Indeed, those calli did not differentiate into ECs, and thus no further experiments were conducted with these non-transgenic cultivars.

### Gene Cloning and Vector Construction

The nucleotide sequences of *GhLEC1*, *GhLEC2*, and *GhFUS3* were obtained from the D subgenome database of *Gossypium. raimondii* by comparing with amino acid sequences of *AtLEC1*, *AtLEC2*, and *AtFUS3* using the tblastn tool. The three genes were then amplified from a full-length cDNA library of CRI24 with specific primers (Table S1). For ectopic expression of *AtWus*, the full-length coding regions (CDS) of the gene was cloned from wild-type *Arabidopsis* (Columbia ecotype) (Table S1). The full-length CDS of *AtWus* was amplified via PCR with specific primers (Table S1) and ligated into vector pMD18-T. After verifying the sequence, each of the *AtWus* fragment and Vector pBI121 was digested with *Bam*H I and *Sac* I. and the *AtWus* fragment was inserted into pBI121. The nucleotide sequences of *GhPIN7*, *GhSHY2*, and *GhARF3* were obtained from the D subgenome database of *G. raimondii* by comparing with amino acid sequences of *AtPIN1*, *AtSHY2*, and *AtARF1* using the tblastn tool.

### RNA Extraction

All calli of CRI24, CRI12, CRI41 and Lu28 cultured for 90 days in NEIM and of 35S:WUS and CK lines cultured for 4 months in EIM were stored at −80°C. We extracted RNA of the above samples using a modified CTAB method [26]. RNA samples with A260/A280 ratios between 1.8 and 2.0 and A260/A230 ratios >1.5 were considered acceptable.

### Quantitative Real Time PCR (QPCR)

Approximately 1 µg total RNA samples were reverse transcribed using the PrimeScript RT reagent kit with gDNA Eraser (Takara). The cDNA templates were diluted three times prior to amplification. The QPCR experiment was conducted according to the guidelines of SYBR Premix Ex Taq™ kit (Takara). QPCR was performed in 96-well plates with a total volume of 20 µL containing 10 µL 2× SYBR Premix Ex Taq™, 6.8 µL PCR-grade water, 2 µL cDNA template, 0.4 µL 50× ROX reference dye I, and 0.4 µL each of forward and reverse primers (10 µM). All QPCRs were run with three technical replicates on an ABI 7900 Real-Time PCR system (Applied Biosystems). The thermal cycling conditions were as follows: an initial denaturation step of 30 s at 95°C, followed by 40 cycles of 95°C annealing for 5 s and 60°C extension for 30 s. The primers used for QPCR are shown in Table S2.

### Scanning Electron Microscopy

Scanning electron microscopy was performed on somatic embryos obtained after 1.5 months culture on SIM. Samples were prefixed at room temperature for 12 h in 2.5% (v/v) glutaraldehyde (phosphate buffer, pH 7.2). After dehydration using a graded ethanol series, samples were dried with a CO_2_ critical-point drying system (HITACHI HCP-2). Subsequently, samples were sputtered with gold dust and observed under a HITACHI S-530 scanning electron microscopy.

### Statistics

The rate of EC = number of EC/number of calli. After 45 days of culture in SIM, we determined the weight of the abnormal embryos in 35S:WUS lines and normal cotyledonary embryos in CK lines with three technical replicates. The average weight of each individual somatic embryo = weight of 10 somatic embryos/10. We conducted t-tests to determine significant differences (p<0.05 or p<0.01, depending on the experiment).

## Results

### Expression of *GhLEC* and *GhFUS3* in Cultivars with Diverse Differentiation Rates

**Figure 1:**
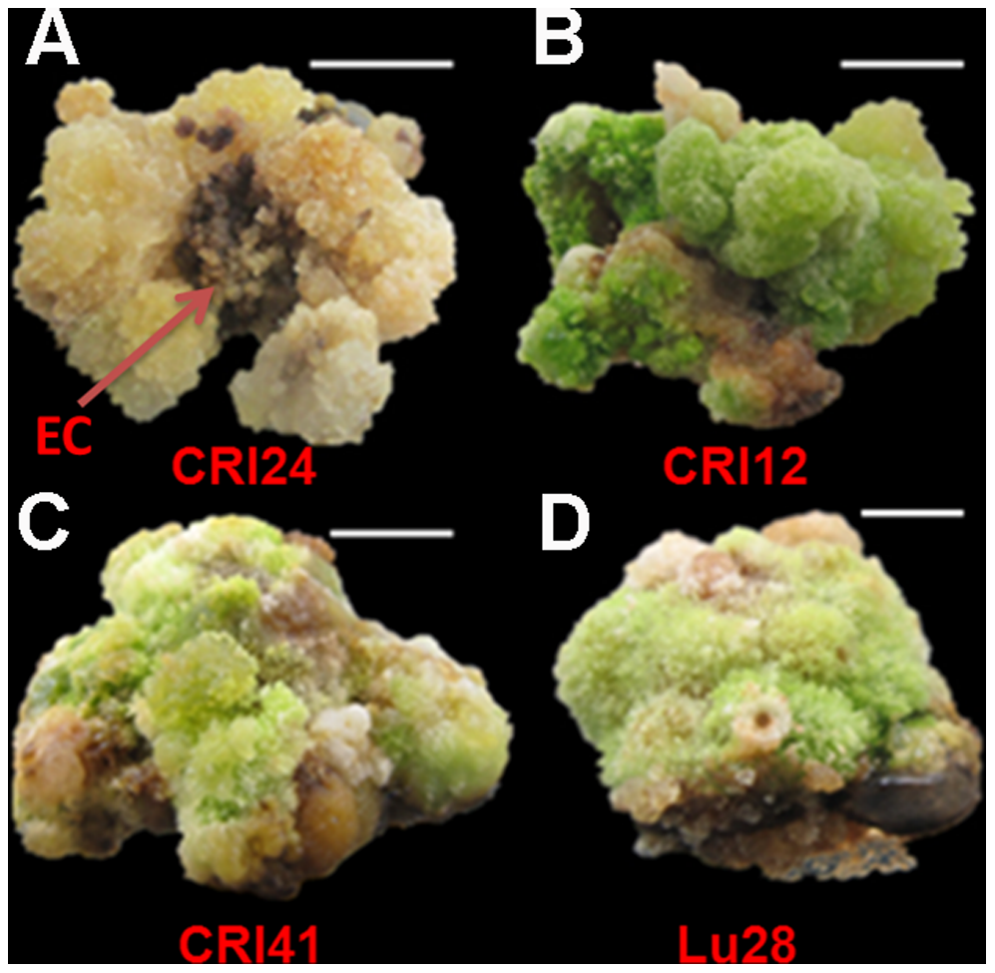
Cotton cultivars have different differentiation rates in EC. After 90 days of culture in NEIM, all calli of CRI24 produced EC, whereas calli of CRI12, CRI41, and Lu28 were dark green and tight and unable to differentiate into EC. Bar, 1

We used four cotton cultivars (*G. hirsutum*) for tissue culture. The EC differentiation rate of CRI24 was 100% but only 0.59% for CRI12, whereas the calli of Lu28 and CRI41 could not form EC (Table 1). After culturing hypocotyl segments for 30 days on NCIM (callus induction medium lacking kanamycin and cefotaxime), the induced calli were transferred onto NEIM (EC induction medium lackling kanamycin and cefotaxime). After 90 days in NEIM, all the CRI24 calli were non-compacted and had differentiated into EC (Figure 1**A**). However, most calli of CRI12 and all calli of CRI41 and Lu28 were compacted, dark green, and did not differentiate (Figure 1**B**–**D**).

**Table 1:** EC induction in four cotton cultivars.

	CRI12 (WT)			CRI24 (WT)			CRI41 (WT)			Lu28 (WT)		
Replication	C^a^	EC^b^	EC rate (%)	C^a^	EC^b^	EC rate (%)	C^a^	EC^b^	EC rate (%)	C^a^	EC^b^	EC rate (%)
1	175	1	0.57	371	371	100	177	0	0.00	209	0	0.00
2	221	3	1.36	204	204	100	303	0	0.00	242	0	0.00
3	286	0	0.00	261	261	100	319	0	0.00	138	0	0.00
Average	682	4	0.59	836	836	100	799	0	0.00	589	0	0.00

a Number of explants forming a callus.

b Number of EC forming from a callus. WT, wild type.

*LEC1*, *LEC2*, and *FUS3* are essential for SE [21], but there are few studies on the roles of *LEC* and *FUS3* in SE in plants other than *Arabidopsis*. Hence, we first isolated the homologs *GhLEC1*, *GhLEC2*, *GhFUS3* in CRI24 [27]. At the amino acid level, these homologs share high sequence similarity with those of in *Arabidopsis* (Figure S1). The results implied that *GhLEC1*, *GhLEC2*, and *GhFUS3* may have functions similar to those of the *Arabidopsis* homologs that control the capacity for SE [21].

**Figure 2:**
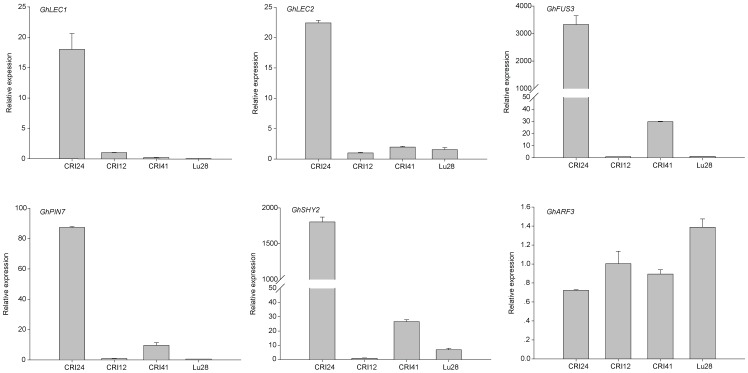
Analysis of gene expression in the non-transgenic calli of the four cultivars.

To investigate the expression of *GhLEC1*, *GhLEC2*, and *GhFUS3* in calli of the four cultivars, we carried out QPCR on calli cultured for 90 days on NEIM. The expression levels of *GhLEC1*, *GhLEC2*, and *GhFUS3* were significantly higher in the calli of CRI24 than in CRI12, Lu28, and CRI41, with barely detectable expression of *GhFUS3* in CRI12 and Lu28 (Figure 2).

### Expression of Genes Involved in Auxin Transport or Signaling Pathways

Auxin plays an important role in SE of cotton [13], [28], but genes involved in auxin transport (*PIN*s) and auxin signaling are not well studied in cotton SE. QPCR analysis of expression levels of these genes in SE used the same samples as for expression patterns of *LECs* and *FUS3*. *GhPIN7* and *GhSHY2* (*AUX/IAA2*) levels in calli of CRI24 were much higher than in the other three cultivars (Figure 2). In contrast, *GhARF3* expression was lower in CRI24 than in other cultivars (Figure 2), suggesting that *GhARF3* expression may be inhibited by the *GhSHY2* gene product. These results indicated that the auxin transport and signaling pathways were more active in calli with high EC differentiation rates than those with low EC differentiation rates.

### Ectopic Expression of *AtWus* Improves EC Induction

**Figure 3:**
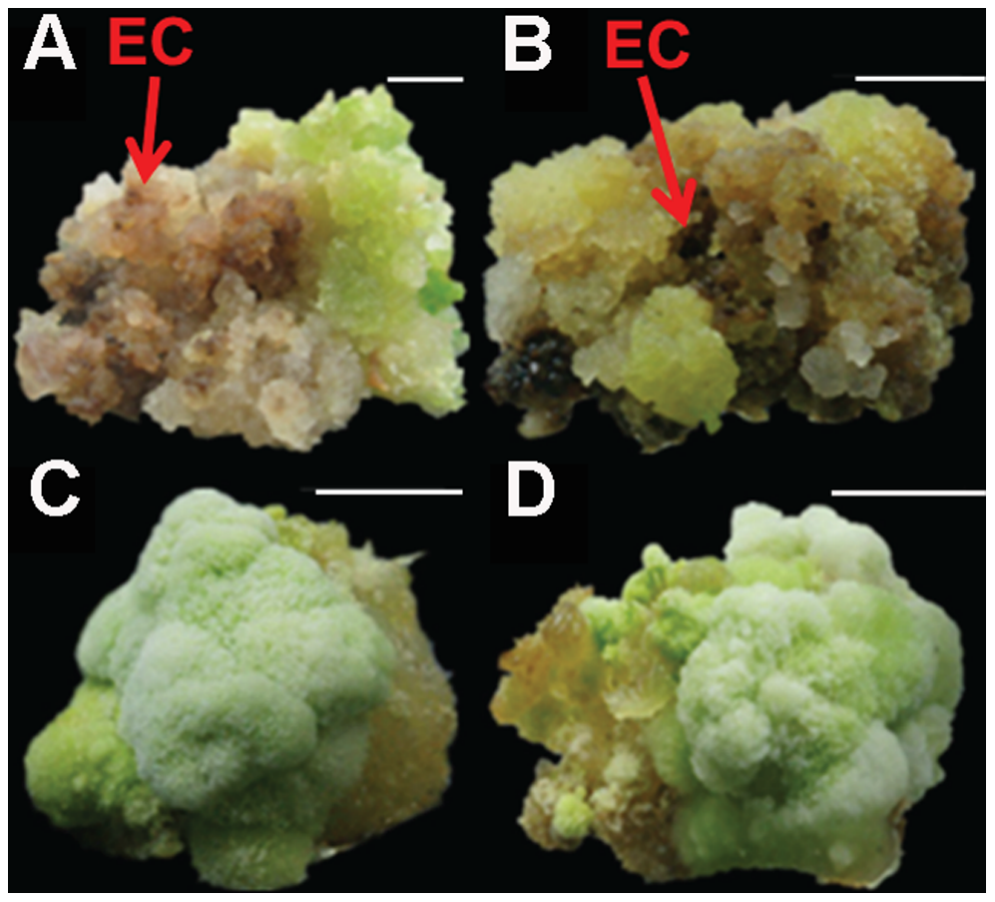
The callus of 35S:WUS and CK cultured for 1.5 months in EIM. **A**, **B:** Calli of 35S:Wus lines at the beginning of EC formation, **C, D:** Calli of CK lines were unable to differentiate. Bar, 1 cm.

CRI12 is one of the most recalcitrant cotton cultivars for plant regeneration via SE. To study the function of *AtWus* in SE, we overexpressed *AtWus* in CRI12 (35S:WUS). An empty vector was used as the control (CK) for parallel transformation. Hypocotyl segments transformed with 35S:WUS or the control CK were cultured on CIM containing kanamycin and cefotaxime to induce resistant calli. One month later, these segments were transferred to fresh CIM. There were no apparent differences between 35S:WUS and CK within the first 2 months. Then, calli that formed on segments were transferred to EIM containing kanamycin and cefotaxime for inducing EC. Calli were transferred to fresh EIM monthly. After culture for 1.5 months on EIM, most 35S:WUS calli were non-compacted and light green, and some began to differentiate into EC (Figure 3**A, B**). However, the CK calli were compact and dark green and did not undergo EC differentiation (Figure 3**C, D**). After culturing for 4 months on EIM, the differentiation rate of 35S:WUS transformants was 47.75% compared with 0.61% for CK (Table 2).

**Table 2:** EC induction in CRI12.

	35S:WUS			Empty vector (CK)		
Replication	C^a^	EC^b^	EC rate (%)	C^a^	EC^b^	EC rate (%)
1	173	88	50.66	151	2	1.32
2	201	94	46.77	114	0	0.00
3	93	41	44.09	223	1	0.45
Average	467	223	47.75**	488	3	0.61

a Number of explants forming callus.

b Number of EC forming from a callus. **p<0.01.

**Figure 4:**
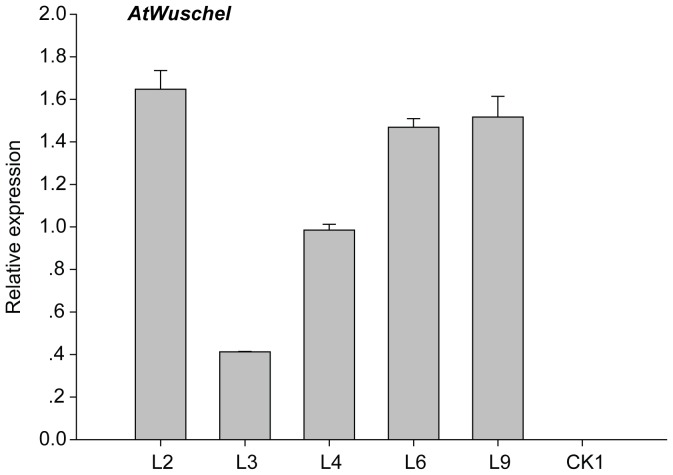
*AtWus* expression in calli of CRI12.

QPCR was used to determine *AtWus* expression level in transformed calli. The calli of transgenic cultures (L2, L3, L4, L6, and L9) cultured for 4 months in EIM were selected for analysis, whereas the calli of CK that did not differentiate into EC served as the control (CK1). The data revealed *AtWus* was overexpressed in 35S:WUS cultures (Figure 4).

### Ectopic Expression of *AtWus* Regulates Auxin Transport and Signal Transduction

**Figure 5:**
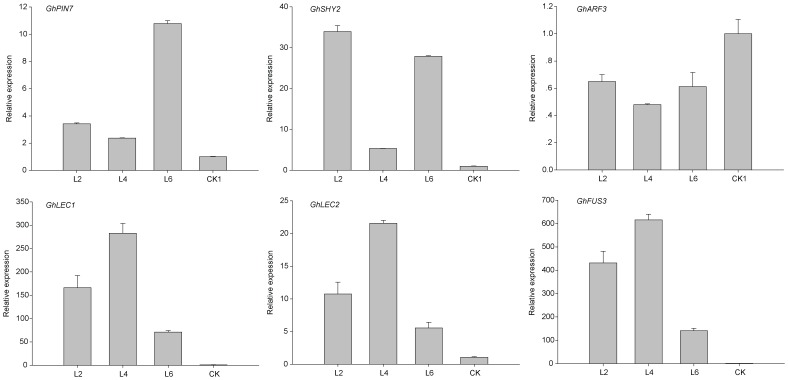
Analysis of gene expression in the transgenic calli carrying *AtWus*.

*AtWus* induction by IAA can regulate *AtPIN* expression during SE in *Arabidopsis* [10]. Hence, the role of *AtWus* in auxin transport and signal transduction in cotton was studied. Transgenic calli of L2, L3, L4, L6, and L9 cultured for 4 months in EIM were analyzed by QPCR, with CK1 calli serving as a control. *GhPIN7* expression was higher in 35S:WUS transformed callus lines than in CK (Figure 5).

Transcript profiling during SE in cotton has been used to establish the association between the auxin signaling pathway and callus differentiation [13]. The above-mentioned data also demonstrated an interaction between the auxin signaling pathway and EC induction whereby *GhSHY2* transcripts were increased in 35S:WUS lines compared with the CK1 line. However, *GhARF3* transcripts were reduced in 35S:WUS, possibly owing to suppression by *GhSHY2* (Figure 5).

### *AtWus* Activates the Expression of *LEC* and *FUS3* during SE in Cotton

*GhLEC1*, *GhLEC2*, and *GhFUS3* were expressed in calli of CRI24 but only barely detected in CRI12. To characterize the regulatory relation between *AtWus* and *LEC*/*FUS3*, we selected L2, L4, and L6 callus lines cultured for 4 months in EIM for further study. QPCR analysis revealed that *GhLEC1*, *GhLEC2*, and *GhFUS3* transcript levels were low in calli of CK1. In calli of L2, L4 and L6, however, higher expression levels of the three genes were detected owing to upregulation by *AtWus* (Figure 5). Hence, *AtWus* promoted the expression of *GhLEC1*, *GhLEC2*, and *GhFUS3* during SE in cotton, similar to *Arabidopsis* [10].

### *AtWus* Overexpression Results in Abnormal Development of Somatic Embryos

**Figure 6:**
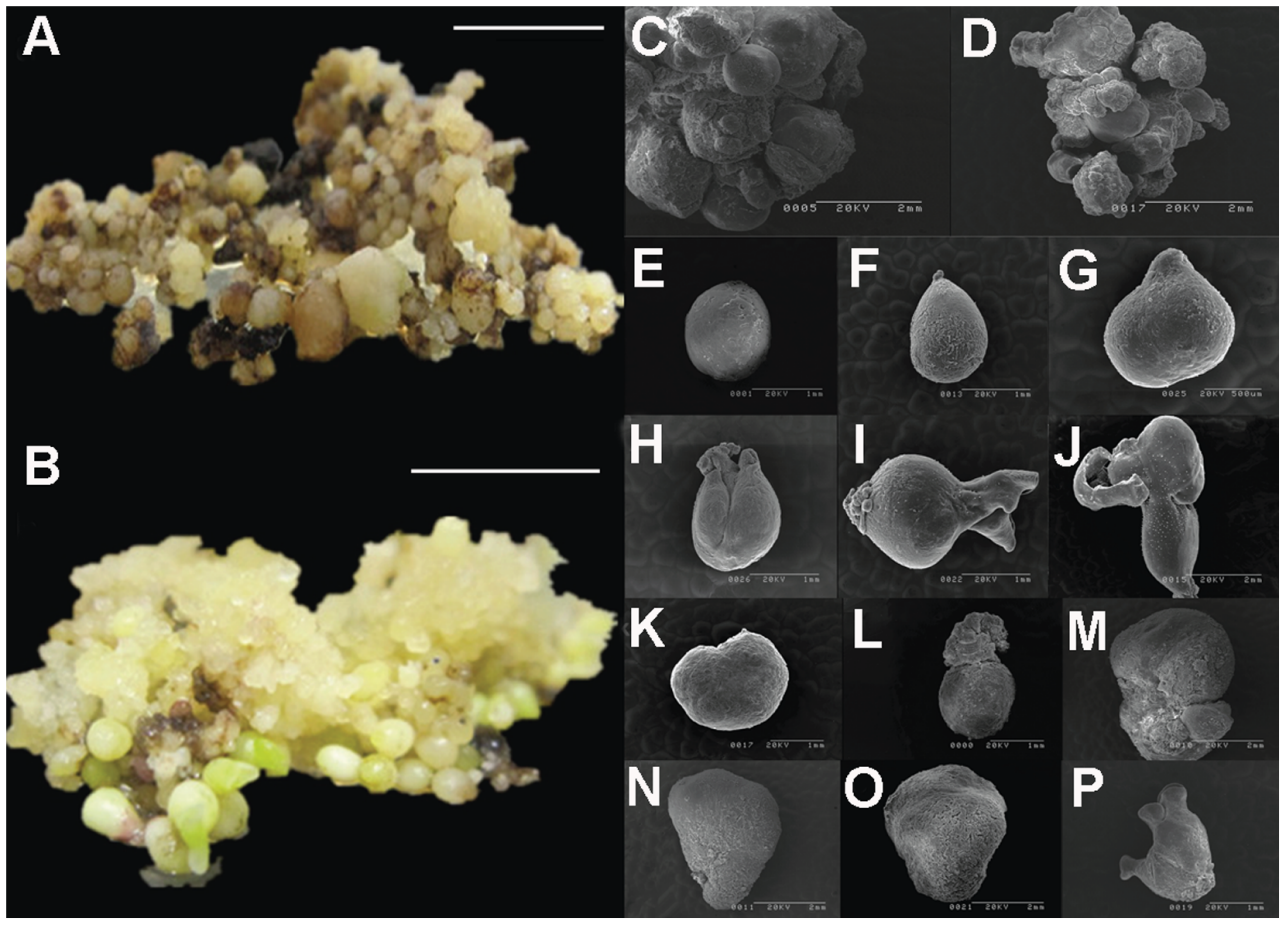
AtWus overexpression results in abnormal development of somatic embryos. **A:** Many abnormal somatic embryos were produced in 35S:WUS lines, and the somatic embryos were inflated and lacked cotyledons. **B:** Formation of normal somatic embryos in CK lines at different stages. Scanning electron microscopy: Holistic perspective of somatic embryos in 35S:WUS lines (**C**) and CK lines (**D**). **E**–**J:** Normal somatic embryos at different stages. **E**, **F:** globular embryo. **G:** heart-shape embryo. **H**–**J:** cotyledonary embryo. **K**–**P:** Abnormal somatic embryos having various appearance. **O:** leaf-like embryo. **P:** multiple-cotyledon embryo. Bar in **A** or **B,** 1 cm.

**Figure 7:**
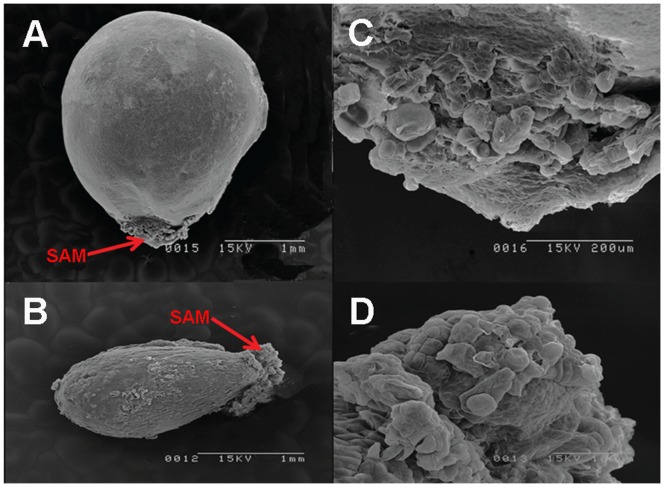
SAM structure of somatic embryos in transgenic lines observed with scanning electron microscopy. **A:** Abnormal somatic embryo of 35S:WUS lines. **B:** Normal somatic embryo of CK lines. **C:** Enlarged image of SAM in **A**. **D:** Enlarged image of SAM in **B**.

For somatic embryo induction, 4-month-old EC of 35S:WUS and CK lines were transferred to SIM containing kanamycin and cefotaxime for somatic embryo induction. ECs were transferred onto a fresh SIM once per month and cultured for 50 days on SIM. EC formed from calli of CK lines grew into normal-looking globular, heart-shaped, and cotyledonary embryos (Figure 6 **B**). However, ECs that developed from 35S:WUS callus lines grew into various abnormal translucent embryos (Figure 6**A**) that could not form the normal-looking cotyledonary embryos except for some leaf-like or multi-cotyledon embryos, although they produced more somatic embryos than CK (Figure 6**C, D**). Scanning electron microscopy was used to investigate the structural abnormalities of these embryos. Somatic embryos from 35S:WUS lines were much larger and heavier than those from CK lines (Figure 6**C**, Table 3). In CK lines, globular, heart-shaped and cotyledonary embryos were clearly observed (Figure 6**E–J**). However, somatic embryos of 35S:WUS lines exhibited several abnormal morphologies, such as leaf-like or multi-cotyledon embryos (Figure 6**K**–**P**). Cotyledons are derived from the shoot apical meristem (SAM) region. SAM cells were arranged in an organized manner in CK somatic embryos (Figure 7**B, D**), but the SAM cells of 35S:WUS lines were disorganized and rather unstructured (Figure 7**A, C**).

**Table 3:** Somatic embryo weight in CRI12.

Replication	35S:WUS (g)	CK (g)
1	0.0138	0.00764
2	0.0124	0.00445
3	0.0181	0.00530
Average	0.0148	0.00580

* p<0.05.

## Discussion

*Wus* is a key gene that controls renewal of stem cells in the apical meristem [29]. However, because *AtWus* promotes the vegetative-to-embryonic transition during SE in *Arabidopsis*, numerous studies have elucidated its functions during SE in several plant species [30]. During preparation of the current report, *AtWus* was shown to promote SE differentiation in *G. hirsutum* Coker cotton [31], a cultivar that easily undergoes SE. However, it is unknown if these results apply to other cotton genotypes. Furthermore, little work has been done on the regulatory role of *Wus* in the auxin signaling pathway during SE.

Here, we examined the role and molecular mechanism of *Wus*-promoted SE in *G. hirsutum*. Cultivar CRI12, a genotype that is difficult to regenerate. We constitutively overexpressed *AtWus* in CRI12 using an *Agrobacterium*-mediated transformation and found that *AtWus* can promote the formation of non-compact light green calli that induce EC easily. The EC differentiation rate was higher in 35S:WUS of CRI12 (47.75%) in comparison with <1% in CK. Hence, *AtWus* is a possible candidate that could promote the nonembryonic-to-embryonic transition during SE in cotton.

Transcript analysis has revealed that the auxin transport and signaling pathways may play a substantive role in EC induction [13]. Our QPCR results showed that *GhPIN*s and *GhSHY*/*GhARF* may regulate the efficiency of callus differentiation into EC. *PINs* play a fundamental role in embryonic auxin distribution in plant embryos [16], and *AtPIN1* is associated with the establishment of auxin gradients during SE in *Arabidopsis*. Previous studies revealed diverse changes in the endogenous auxin levels when EC was induced [13]. The antisense cDNA of *AtWus* suppresses *PIN*s during SE in *Arabidopsis* [10]. When auxin levels are low, *SHY* (*AUX/IAA*) expression is induced and certain *ARF* activity is repressed, but at high auxin levels SHY is degraded by SCF^TIR1/AFBs^ and the *ARF* inhibitory action is abolished [17], [19]. Our results revealed enhanced *GhSHY2* expression and repressed *GhARF3* expression in CRI12 calli overexpressing *AtWus*. However, *AtWus* is unable to modify the amount of auxin in cotton calli [31]. Hence, *AtWus* may play an important role in upregulating *GhPIN*s to redistribute auxin gradients, which may alter expression patterns of *SHY*-*ARF* at low levels of auxin.

*LEC1*, *LEC2*, and *FUS3* are crucial for SE. The capacity of SE is almost completely repressed in double and triple mutants of the three genes, indicating that *LEC* genes may function downstream of endogenous auxin-induced SE in *Arabidopsis* [21], [23]. In our present study, *GhLEC1*, *GhLEC2*, and *GhFUS3* transcript levels were extremely low in callus that was unable to differentiate into EC, but levels were high in callus producing EC. Hence, *AtWus* positively regulated *LECs* and *FUS3*. In *Arabidopsis*, WUSCHEL and PGA37/MYB118 promote SE and activate the expression of *LEC1*, *LEC2*, and *FUS3* [32]. In our study, *GhLEC1*, *GhLEC2*, and *GhFUS3* were also upregulated in callus of 35S:WUS lines. These results suggest that *AtWus* may alter *PIN* expression, which leads to the establishment of new auxin gradients in the callus. Subsequently, a new auxin response was formed and stimulated *GhLEC1*, *GhLEC2*, and *GhFUS3* in the callus of CRI12. *AtWus* may provoke the ability of differentiation in the callus by reactivating *GhLECs* and *GhFUS3* expression through auxin transport and signaling mechanisms.

Although *AtWus* improved EC induction, the observed abnormal somatic embryos were an unexpected consequence of *AtWus* overexpression and prevented seedling generation. *AtWus* expression is limited to the SAM because auxin accumulates in the cotyledon primordial cells during somatic embryo development in *Arabidopsis* [10]. Therefore, ectopic expression of *Wus* could cause loss of expression specificity in somatic embryos, leading to asymmetric growth. In *Arabidopsis*, *LEC1* and *FUS3* may control multiple aspects of seed development [33]. Constitutive expression of *LEC1* leads to occasional formation of somatic embryo like structures [34]. Therefore, constitutive expression of *AtWus* in CRI12 may have led to constitutive expression of *GhLEC1*, *GhLEC2* and *GhFUS3* in embryos, and then the expression of *GhLECs* and *GhFUS3* may have resulted in the observed abnormal embryos and failure of seedling regeneration. Using inducible promoters such as the estradiol-inducible promoter [35] rather than the 35S promoter during SE in cotton may avoid abnormal embryo formation. Estradiol could be added into the CIM and EIM for EC induction with subsequent transfer of ECs onto SIM without estradiol for normal embryo development. This promoter has been successfully applied for SE in several species [30].

Cotton is an important source of textile fiber and edible oil, but cotton yield is adversely affected by abiotic or biotic stresses [36]. Therefore, efforts to improve cotton resistance against such stresses by genetic modification may play a vital role in efforts to increase production. *Agrobacterium*-mediated transformation via SE has been the most popular transgenic technology in cotton. Most genotypes cannot undergo EC induction or have low rates of differentiation, although many of those recalcitrant genotypes have certain positive agronomic characters [37]. Thus, the difficulty of EC induction always restricts the application of transgenic breeding and *in vitro* regeneration in additional cultivars. For example, CRI12 used to be an important elite cultivar widely cultured in China for its disease resistance, high yeild and superior fiber quality. However, SE production in this cultivar is not easy, making it difficult to improve traits using transgenic technology. In our study, the introduction of *AtWus* into the recalcitrant cotton cultivars enhanced their somatic embryogenesis. With this foundation established, we may now construct a vector to overexpress *AtWus* with an estradiol-inducible promoter and transfer it into CRI12 or other cultivars. This will require a simple addition of estradiol to CIM and EIM to ensure EC induction with high frequency and production of transgenic seedlings. This will then enable us to improve the rate of cotton transformation for many foreign genes or cotton genes. Such transgenic plants can be used directly as germplasm for cotton breeding. This protocol will achieve our goals of creating more germplasm resources that facilitate SE and expand the scope of transgenic breeding in more cultivars.

## Supporting Information

Figure S1 Characterization of *GhLEC1*, *GhLEC2* and *GhFUS3*.
(TIF)

Table S1 Specific primers for cloning *GhLEC1*, *GhLEC2*, *GhFUS3* and *AtWuschel*.
(XLSX)

Table S2 Primers used for quantitative real time PCR.
(DOCX)
