# Supplementary figures and images for: AtWuschel Promotes Formation of the Embryogenic Callus in Gossypium hirsutum
Source: PLoS One. 2014 Jan 31;9(1):e87502. doi: 10.1371/journal.pone.0087502 (PMC3909107; doi:10.1371/journal.pone.0087502)

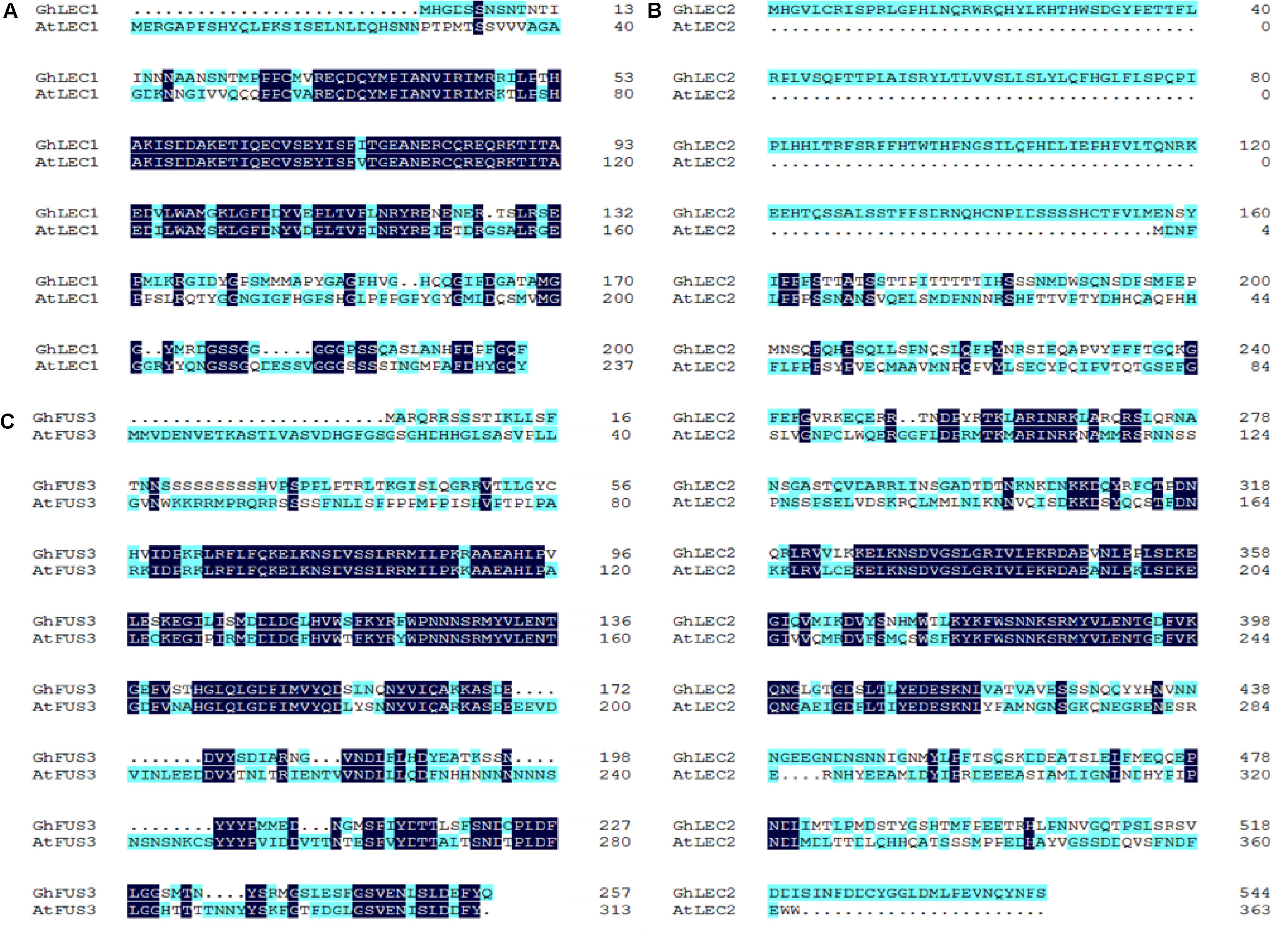

Supplement: Figure S1 — Characterization of GhLEC1, GhLEC2 and GhFUS3. (TIF) [file pone.0087502.s001.tif]
