# Supplementary material for: AtWuschel Promotes Formation of the Embryogenic Callus in Gossypium hirsutum
Source: PLoS One. 2014 Jan 31;9(1):e87502. doi: 10.1371/journal.pone.0087502 (PMC3909107; doi:10.1371/journal.pone.0087502)
Supplement: Table S2 — Primers used for quantitative real time PCR. (DOCX) [file pone.0087502.s003.docx]

**Table S1.** Primers used for quantitative real time PCR

| Genes | Primers (5’-3’) |
| --- | --- |
| *Histone3* | TCAAGACTGATTTGCGTTTCCA  GCGCAAAGGTTGGTGTCTTC |
| *AtWuschel* | TATGATGGCGGCTAACGA  GATGATAGAGATGATGGTCTTGG |
| *GhLEC1* | TCTATGATGATGGCACCTTATGG  GCTTGTGACGATGGTCCACC |
| *GhLEC2* | CCATCCATTCCTCCTCAAACA  CAGGCTCTGGTTAGGTGACAAA |
| *GhFUS3* | GGGTATTTCCTTACAGGGTCGG  GAGTATCATTCTTCGGAGGGAGC |
| *GhPIN7* | TCAGTCTTGGTCTGTTTATGGCAT  AGCAATGGAAGCAGCAGCC |
| *GhSHY2* | AAGATTAGGGTTGCCAGGC  TCCCACCACACTCACTTTCAC |
| *GhARF3* | GAGCAGGAGAAACAGCCTCGTA  CCACCGACCTACAAAGAGCAA |

Each top sequence is the forward primer, and each bottom sequence is the reverse primer.
